# Immunotherapy in Prostate Cancer: From a “Cold” Tumor to a “Hot” Prospect

**DOI:** 10.3390/cancers17071064

**Published:** 2025-03-21

**Authors:** Whi-An Kwon, Jae Young Joung

**Affiliations:** 1 Department of Urology, Hanyang University College of Medicine, Myongji Hospital, Goyang 10475, Republic of Korea; 2 Department of Urology, Urological Cancer Center, National Cancer Center, Goyang 10408, Republic of Korea

**Keywords:** androgen receptor, cancer vaccines, immune checkpoint inhibitors, immunosuppressive microenvironment, immunotherapy, microsatellite instability, prostate cancer, tumor microenvironment

## Abstract

Immunotherapy has shown limited efficacy in prostate cancer, largely due to low tumor immunogenicity, sparse tumor-infiltrating lymphocytes, and a suppressive microenvironment. Recent therapeutic strategies aim to boost immune responses and counteract immunosuppressive factors through interventions such as immune checkpoint inhibitors, immunogenic cell death-inducing therapies, and the targeted blockade of pathways like that of transforming growth factor-β. Vaccine-based approaches, potent immune adjuvants, and engineered chimeric antigen receptor (CAR) T cells are also being investigated to overcome local immune inhibitory signals. Advancements in imaging, multi-omic profiling, and liquid biopsies offer promising avenues for real-time monitoring, better patient selection, and precision treatment. This review provides an overview of the key immunosuppressive features of prostate cancer, current immunotherapeutic modalities, and emerging strategies to transform “cold” tumors into more responsive “hot” targets. By integrating these approaches, we may achieve more durable clinical benefits for patients with advanced or metastatic prostate cancer.

## Simple Summary

Prostate cancer, often considered immunologically “cold”, can respond to immunotherapy when the tumor microenvironment is effectively reprogrammed. Combination strategies that integrate checkpoint inhibitors, chimeric antigen receptor T-cell therapies, or cancer vaccines with chemotherapy, hormonal therapy, or radiotherapy have shown promise by boosting antigen presentation and overcoming immunosuppression. Emerging biomarkers such as microsatellite instability and DNA repair defects help to identify patients most likely to benefit from these approaches. Ongoing research focuses on refining personalized treatment plans, managing toxicity, and innovating clinical trial designs for more durable disease control.

## 1. Introduction

Prostate cancer is one of the most common cancers in men worldwide and is particularly challenging as it progresses to advanced or metastatic stages, leading to high mortality rates [1]. Although surgical intervention, radiation therapy, and hormone therapy can be effective for early localized tumors, many patients inevitably develop castration-resistant prostate cancer (CRPC), for which current treatments offer limited long-term survival. Consequently, there is a pressing need for innovative therapeutic approaches to achieve durable disease control. In recent years, immunotherapy has revolutionized the treatment of melanoma, lung cancer, and urothelial carcinoma, sparking considerable interest as to whether similar strategies can be applied to prostate cancer. Yet prostate cancer is often characterized as “cold”, meaning that it has few tumor-infiltrating lymphocytes, low neoantigen expression, and an abundance of immunosuppressive cells, making the tumor environment less responsive to immune-based interventions. Overcoming these barriers calls for a deeper understanding of underlying mechanisms and more sophisticated treatment combinations designed to convert “cold” tumors into those more receptive to immunotherapy [2,3].

The immunosuppressive milieu in prostate cancer includes regulatory T cells, tumor-associated macrophages, and myeloid-derived suppressor cells that effectively inhibit immune activation [4]. Nonetheless, the initial success of sipuleucel-T, the first therapeutic cancer vaccine approved for prostate cancer, demonstrates that immunotherapy can play a meaningful role if the tumor microenvironment (TME) and patient selection are suitably managed [5]. Although a variety of immunotherapeutic strategies, such as immune checkpoint inhibitors (ICIs), chimeric antigen receptor (CAR) T-cell therapy, and cancer vaccines, have shown promise, their stand-alone efficacy in prostate cancer is often limited [4]. Recent approaches have focused on inducing immunogenic cell death (ICD)—a form of cell death that not only eliminates tumor cells but also triggers an immune response through the release of damage-associated molecular patterns (DAMPs) and tumor antigens—in tumor cells through chemotherapy, radiotherapy, photothermal therapy, and other methods that release tumor antigens and damage-associated molecular patterns (DAMPs), thereby activating antigen-presenting cells and amplifying T-cell infiltration [6]. Novel biomaterial-based platforms and nanomaterials further enhance this process by modulating the TME and improving antigen presentation [7].

Patient-specific genomic features, including programmed cell death ligand 1 (PD-L1) expression, microsatellite instability (MSI), DNA repair gene mutations, and tumor mutational burden (TMB), are increasingly central to optimizing immunotherapy for prostate cancer. Such biomarkers help identify patients who are most likely to benefit from ICIs, CAR T-cell therapy, or cancer vaccines [8]. Although PD-L1, MSI, and TMB have shown promise as predictive biomarkers for immunotherapy response in various cancers, their specific roles in prostate cancer remain under active investigation and have not yet been fully prospectively validated [9].

When these immunotherapies are combined with conventional treatments, such as radiation, chemotherapy, or hormone therapy, synergistic effects can significantly enhance immune responses. Indeed, some patients with prostate cancer have shown pronounced benefits from this multimodal approach, reinforcing the value of personalized treatment plans [10,11]. It is important to note that immunotherapy outcomes differ between primary prostate tumors and castration-resistant prostate cancer (CRPC), with primary lesions typically exhibiting a more profoundly “cold” immune microenvironment, while CRPC—especially when combined with androgen deprivation or other modulatory treatments—may show a modest increase in T-cell infiltration and immunogenicity, thus warranting tailored therapeutic approaches [12].

Although prostate cancer is typically regarded as an immunologically “cold” tumor, recent discoveries show that reshaping the TME can unlock its immunotherapy potential [13,14]. Unlike purely descriptive reviews, this paper highlights practical strategies for converting “cold” tumors into “hot” tumors, placing special emphasis on how combination therapies and biomarker-driven patient selection can improve clinical outcomes. By exploring these methods, this review highlights the pathways for maximizing the effectiveness of immunotherapy in prostate cancer and offers insights relevant to other solid tumors facing similar challenges. This review includes a thorough examination of the immunosuppressive features of prostate cancer, the latest immunotherapeutic developments, and the refinement of treatment through synergistic combinations, all aimed at guiding future research and improving patient care.

## 2. TME and the Immunological Landscape of Prostate Cancer

In recent years, the interplay between prostate cancer pathobiology and the host immune response has garnered increasing attention. Despite major strides in understanding tumor immunology across diverse malignancies, prostate cancer remains a paradigm of a “cold tumor”, characterized by a paucity of tumor-infiltrating lymphocytes and a microenvironment dominated by immunosuppressive cells and soluble factors [15]. We examine the molecular underpinnings of prostate cancer’s immunological landscape and integrate historical developments, contemporary findings, and future perspectives. Through a critical appraisal of existing evidence and conflicting reports, we aim to highlight methodological and clinical nuances that frame ongoing debates and innovative research directions.

### 2.1. Molecular Pathophysiology

#### 2.1.1. Androgen Receptor (AR) Signaling and Historical Context

The seminal mid-20th-century discoveries by Huggins and Hodges demonstrating that orchiectomy or estrogen therapy could regress prostate tumors laid the cornerstone for androgen deprivation therapy (ADT)—a mainstay of prostate cancer treatment for decades. These pioneering studies not only underscored hormonal control over prostate growth but also exposed avenues for therapeutic manipulation [16].

Mechanistically, the androgen receptor (AR) is activated by testosterone or dihydrotestosterone, subsequently translocating to the nucleus to drive the transcription of genes crucial for cell proliferation and survival. Prostate cancer cells exploit this pathway, leveraging AR signaling to sustain relentless growth. Although first-line ADT effectively induces tumor regression in most hormone-sensitive cases, progression to castration-resistant prostate cancer (CRPC) frequently occurs, highlighting the tumor’s adaptive resistance capabilities [17].

Immunologically, short-term ADT has been shown to enhance T-cell infiltration into prostate tumors, temporarily establishing an immunologically active tumor microenvironment. However, this immunologic benefit tends to be transient due to tumor development of immune escape mechanisms. These include increased expression of immune checkpoint molecules such as PD-L1, T-cell exhaustion, and the accumulation of immunosuppressive cell populations, notably regulatory T cells (Tregs) and myeloid-derived suppressor cells (MDSCs), which ultimately diminish the antitumor immune response [18,19].

To overcome this limitation, recent studies have explored combination strategies aimed at prolonging the transient immunogenic window induced by ADT and sustaining durable antitumor immunity. Clinical investigations combining ADT with antigen-specific vaccines have shown promising outcomes. For example, combining ADT with a DNA vaccine targeting the androgen receptor (pTVG-AR) resulted in increased AR-specific CD8+ T-cell infiltration, effectively delaying progression to CRPC. Nonetheless, these studies emphasize the need for additional interventions to mitigate or modulate the emergence of immunosuppressive cell populations following ADT [20].

Similarly, clinical trials evaluating ADT in combination with GVAX—a GM-CSF-secreting whole-cell vaccine—and low-dose cyclophosphamide have demonstrated enhanced CD8+ T-cell infiltration. Yet this regimen simultaneously induces immunosuppressive responses, underscoring the complexity of interactions between hormonal therapies and immunomodulatory approaches [19]. Thus, while combining ADT with immune-based treatments offers significant therapeutic potential, the precise mechanisms underpinning their synergy remain incompletely delineated, necessitating further investigation through rigorous and standardized clinical trials.

#### 2.1.2. Transition to CRPC

CRPC emerges through multiple adaptations, including AR gene amplification, point mutations, and the generation of splice variants such as AR-V7, all of which circumvent conventional ADT. Additionally, some tumors exploit intratumoral androgen synthesis, further sustaining AR signaling under systemic castration. From an immune standpoint, heightened AR activity can restrict cytotoxic T-cell function via shifts in chemokine gradients and the induction of checkpoint molecules such as PD-L1 [21].

Although newer-generation AR pathway inhibitors (e.g., enzalutamide and abiraterone) have been shown to confer survival benefits in randomized trials, their immunological impacts are less consistent. Some evidence suggests a temporary enhancement in tumor immunogenicity, manifested as increased T-cell infiltration or altered cytokine profiles; however, this improvement is neither universal nor long-lasting [21]. Methodological challenges, including small patient cohorts and underpowered phase I/II trials, have prompted efforts to elucidate these effects. Thus, large-scale biomarker-driven studies are essential to determine how best to integrate hormonal blockades with immunotherapies to sustain T-cell activity and overcome tumor escape mechanisms [22,23].

### 2.2. Prostate Cancer as a “Cold Tumor”: Challenges in Immunotherapy

**Figure 1:**
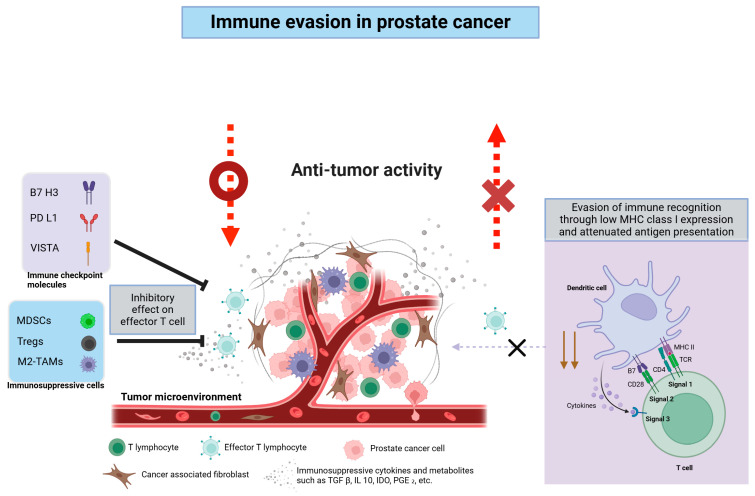
Immunosuppressive tumor microenvironment (TME) of prostate cancer. Schematic illustrating key immune evasion mechanisms in prostate cancer. Prostate cancer cells and cancer-associated fibroblasts release immunosuppressive factors (e.g., TGF-β, IL-10) and express immune checkpoints (e.g., PD-L1, B7-H3, VISTA), thereby suppressing effector T-cell function. MDSCs, Tregs, and M2-TAMs further weaken the antitumor response, while reduced MHC class I expression hampers tumor cell recognition by T cells. Abbreviations: B7-H3, B7 homolog 3; IL-10, interleukin-10; M2-TAM, M2-type tumor-associated macrophage; MDSC, myeloid-derived suppressor cell; MHC, major histocompatibility complex; PD-L1, programmed death ligand 1; TGF-β, transforming growth factor-beta; Treg, regulatory T cell; VISTA, V-domain Ig suppressor of T-cell activation. Created with BioRender.com.

The classification of prostate cancer as a “cold” tumor reflects its sparse lymphocytic infiltration and overall low immunogenicity—a stark contrast to “hot” tumors such as melanoma and non-small cell lung cancer, which often respond favorably to immune checkpoint inhibitors (ICIs) [22]. In immunotherapy, the concepts of “cold” and “hot” tumors are crucial for understanding immune responsiveness. “Hot” tumors typically exhibit high T-cell infiltration, PD-L1 overexpression, a high tumor mutational burden (TMB), and a strong inflammatory phenotype, thereby making them more responsive to ICIs [24]. Conversely, “cold” tumors, like most prostate cancers, demonstrate low or absent T-cell infiltration, low PD-L1 expression, low TMB, and an immune desert or exclusion phenotype, which collectively contribute to their poor response to ICIs [22]. In prostate cancer, immune evasion arises from multiple factors, including limited T-cell infiltration, abundant immunosuppressive cells, immunosuppressive soluble mediators, and a low neoantigen burden. Each of these factors promotes a formidable barrier to antitumor immunity [2,24,25]. Importantly, immune status in prostate cancer can vary, with some patients displaying “hot” characteristics despite the general “cold” nature of the disease [26]. Assessment methods for immune status include immune scoring (evaluating CD3+ and CD8+ T-cell density and distribution), PD-L1 expression analysis, TMB measurement, cytokine profiling (e.g., interferon-γ, interleukins, TNF), and detection of tumor-infiltrating lymphocytes (TILs) [2]. Figure 1 illustrates the key cellular and molecular mediators that constitute the immunosuppressive tumor microenvironment (TME) of prostate cancer, visually complementing the discussion of these immune evasion mechanisms. Applying these assessment criteria to prostate cancer is essential for evaluating individual patients’ immune statuses and for developing tailored immunotherapy strategies to optimize treatment outcomes [27].

#### 2.2.1. Lack of Tumor-Infiltrating Lymphocytes

Single-cell RNA sequencing and immunophenotypic analyses have revealed a striking paucity of T cells within prostate tumors, where the limited CD8+ T lymphocytes predominantly exhibit an exhausted or tissue-resident phenotype, in stark contrast to the robust effector T-cell expansion observed in melanoma or lung cancer. This immunologically “deserted” environment is maintained by several convergent mechanisms, including the upregulation of co-inhibitory molecules, whereby prostate cancer cells express PD-L1 and other ligands that interact with T-cell checkpoints such as PD-1 and CTLA-4, leading to T-cell exhaustion [28,29]. In addition, tumor-derived exosomes enriched with KLK3 (prostate-specific antigen) or interleukin-8 contribute to the suppression of T-cell cytotoxicity through mechanisms involving metabolic reprogramming and direct inhibitory signaling [29,30]. Moreover, emerging evidence indicates that androgen receptor (AR) signaling within CD8+ T cells diminishes their effector function and responsiveness to PD-1 blockade, although the inhibition of AR signaling in these lymphocytes can partially restore their activity [28]. Clinically, while short-term androgen deprivation therapy (ADT) may transiently enhance T-cell trafficking into the TME, achieving sustained and effective T-cell reactivation likely requires additional therapeutic strategies, such as checkpoint inhibitors, tumor vaccines, or radiotherapy, all of which have the potential to upregulate antigen presentation and promote epitope spreading [2,5].

#### 2.2.2. Abundant Immunosuppressive Cells

The immunosuppressive milieu in prostate cancer is further reinforced by tumor-associated macrophages (TAMs), myeloid-derived suppressor cells (MDSCs), and regulatory T cells (Tregs). As summarized in Table 1, prostate cancer cells recruit various immunosuppressive populations—including Tregs, MDSCs, and TAMs—while also upregulating soluble factors such as indoleamine-2,3-dioxygenase (IDO) and transforming growth factor (TGF)-β, together creating a profoundly “cold” TME that limits robust antitumor immunity [25,31]. TAMs, which are often polarized toward the M2 phenotype, secrete CCL5/CCL6 and exosomes enriched with miR95, fostering tumor proliferation and metastasis [32]. In parallel, MDSCs produce arginase 1, indoleamine-2,3-dioxygenase (IDO), and reactive oxygen species (ROS), which directly inhibit T-cell activation and promote Treg expansion [31,33]. Tregs themselves, through perforin/granzyme release or inhibitory cytokines such as transforming growth factor (TGF)-β, IL-10, and IL-35, provide an additional layer of immunosuppression [34]. Notably, the coexistence of these cell types creates robust feedback loops. For instance, TAM-derived signals can recruit and activate MDSCs, which in turn promote Treg differentiation, culminating in a circuit that thwarts cytotoxic T-cell infiltration and function. This complex tapestry underscores why single-agent immunotherapies targeting one suppressive cell type (e.g., colony-stimulating factor 1 receptor [CSF-1R] inhibition to reduce TAMs) frequently yield transient or modest results, highlighting the need for multimodal regimens that disrupt multiple immunosuppressive networks [25].

#### 2.2.3. Soluble Factors in the TME

In addition to cellular mediators, the prostate TME is replete with soluble factors such as cytokines, chemokines, and metabolites, which collectively reinforce an immunosuppressive state. ROS, nitric oxide, and arginase impede T-cell proliferation, while TGF-β and IL-10 drive Treg differentiation. These cytokine networks generate potent immunosuppressive loops, wherein Tregs and MDSCs expand in response to tumor-derived signals and reciprocally bolster tumor-induced immunosuppression [2,5]. Moreover, prostate tumors often consume nutrients (e.g., glucose and amino acids) that are vital for T-cell fitness and generate metabolic byproducts such as adenosine, kynurenine, lactate, and additional ROS, all of which hinder T-cell effector functions. Prostaglandin E2 (PGE2) is notably elevated in prostate cancer and significantly shapes local chemokine gradients, limiting T-cell infiltration and favoring MDSC recruitment. These combined effects underscore why neutralizing or modifying the metabolic environment (e.g., via IDO inhibitors or adenosine pathway blockade) can be critical for reinvigorating T-cell responses [2,35].

#### 2.2.4. Tumor Neoantigens and Low Immunogenicity

The immunogenic potential of prostate cancer is often constrained by its low mutational burden (approximately 0.9%), which translates into fewer neoantigens. This limitation makes it challenging for the immune system to distinguish tumor cells as “non-self”. Although prostate cancer cells express certain tissue-restricted antigens (e.g., PSA, prostate-specific membrane antigen [PSMA], and prostatic acid phosphatase [PAP]), these antigens alone rarely elicit sufficiently robust T-cell responses to overcome entrenched immunosuppressive pressures [5].

Hormonal dependence further blunts ICD because ADT-induced apoptosis does not consistently release the danger signals required for robust T-cell priming. Nonetheless, some patients harbor tumors with MSI-H or mismatch repair deficiencies (dMMR), presenting higher mutational burdens and a potentially more favorable milieu for immunotherapy. Early phase trials have shown promising responses to checkpoint blockade in these molecularly selected subsets; however, larger confirmatory studies are warranted [5].

### 2.3. Key Immune Evasion Mechanisms

Prostate cancer employs multiple evasion strategies, including the downregulation of major histocompatibility complex class I, impaired antigen processing (TAP1/2 deficiencies), and the upregulation of immune checkpoints (PD-L1, B7-H3, and VISTA). These alterations converge in a hostile metabolic environment rich in PGE2, IDO, and adenosine, which undermines T-cell fitness [2,5]. Vaccine-induced immune responses in prostate cancer often fail to yield significant clinical benefits due to a series of interconnected immune escape mechanisms. Prostate cancer cells commonly downregulate MHC class I and suppress antigen-processing genes, impairing cytotoxic T-cell recognition [36]. In addition, the tumor microenvironment secretes immunosuppressive factors—such as TGF-β, adenosine, and IL-10—that further inhibit antitumor immunity, while the presence of regulatory T cells and myeloid-derived suppressor cells compounds this suppression [2]. Nearly 90% of patients also display immunological ignorance, failing to activate antigen-specific immune responses [37], and the simultaneous overexpression of immune checkpoint molecules like CTLA-4, PD-1, and DcR3 diminishes the effectiveness of single-agent therapies [37]. Together, these mechanisms underscore the need for multi-targeted, personalized strategies to overcome resistance and improve immunotherapeutic outcomes in prostate cancer.

**Figure 2:**
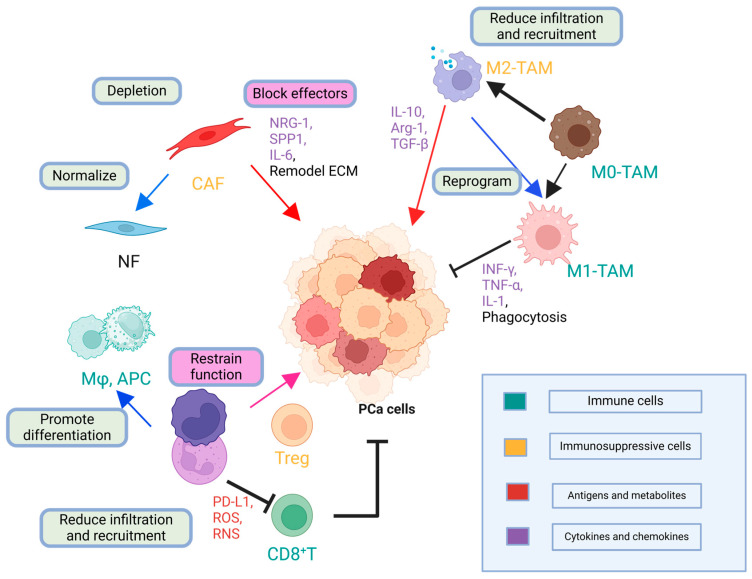
Immune evasion mechanisms and immunosuppressive network in prostate cancer. Centered around PCa cells, cancer-associated fibroblasts (CAFs) release signals (e.g., NRG-1, SPP1, IL-6) and remodel the extracellular matrix, while normal fibroblasts (NFs) may be normalized or depleted. Immune cells—macrophages (Mϕ) and antigen-presenting cells (APCs)—can polarize into M1 or M2 tumor-associated macrophages (TAMs). M2-TAMs release immunosuppressive mediators (IL-10, Arg-1, TGF-β), whereas M1-TAMs produce proinflammatory cytokines (IFN-γ, TNF-α, IL-1). Regulatory T cells (Tregs) also restrain cytotoxic function, while CD8+ T cells are inhibited by PD-L1, reactive oxygen species (ROS), and reactive nitrogen species (RNS, such as nitric oxide and peroxynitrite). Targeting Tregs or M2-TAMs can restore antitumor immunity. Overall, the figure suggests interventions—including CAF depletion/normalization, M2-to-M1 reprogramming, and immune checkpoint blockade—to shift the TME toward effective tumor eradication. Created with BioRender.com.

Despite the transformative effect of checkpoint inhibitors on cancers such as melanoma, the results in prostate cancer have been variable, raising questions about whether checkpoint blockade itself is suboptimal or simply requires better tumor priming. Combination approaches, such as pairing immunotherapy with focal radiotherapy, oncolytic viruses, or tumor vaccines, appear to be more promising because they enhance T-cell infiltration and diversify the repertoire of tumor antigens. However, trial heterogeneity, including disease stage, patient selection, and endpoints (objective response vs. overall survival), complicates direct comparisons. A balanced perspective acknowledges that, while single-agent checkpoint blockade has yielded modest outcomes in prostate cancer, rationally combined therapies and robust patient stratification may unlock greater clinical benefits [38,39]. Figure 2 illustrates the immune evasion mechanisms and interlinked immunosuppressive networks in prostate cancer, encompassing checkpoint molecule upregulation, metabolic reprogramming, and immunosuppressive cell recruitment.

**Table 1:** Major immunosuppressive cells and molecules in the prostate cancer TME.

Immunosuppressive Cell/Molecule	Key Factors	Immunosuppressive Mechanisms	Clinical Significance	References
Tregs	TGF-β, IL-10	Suppress T-cell function	Poor prognosis	[4,34]
MDSCs	Arginase-1, IDO	Impair antigen presentation	Therapeutic target	[31,33]
TAMs (M2 type)	CCL5, TGF-β	Promote tumor growth	High density = worse outcomes	[32]
PD-L1/PD-L2	PD-L1, PD-L2	Induce T-cell exhaustion	Resistance marker	[38,40]
IDO	Tryptophan	Inhibit T-cell growth	Potential combo target	[35]
TGF-β	TGF-β cytokine	Promote Treg development	Potential therapeutic target	[2,4]
Adenosine	CD39/CD73	Suppress immune activity	Novel therapeutic pathway	[2]

Abbreviations: Arg-1, arginase-1; CCL5, C-C motif chemokine ligand 5; IDO, indoleamine 2,3-dioxygenase; IL-10, interleukin-10; MDSC, myeloid-derived suppressor cell; PD-L1, programmed death ligand-1; PD-L2, programmed death ligand-2; TAM, tumor-associated macrophage; TGF-β, transforming growth factor-beta; Treg, regulatory T cell.

### 2.4. Prostate Cancer-Specific Antigens and Therapeutic Targets

Prostate cancer presents with unique antigens (PSA, PSMA, and PAP) that have spurred the development of antigen-specific vaccines and CAR T-cell therapies. These targeted approaches leverage tumor selectivity to minimize off-target toxicity. Nonetheless, antigen downregulation remains a risk factor because prolonged immune pressure can drive tumor cells to diminish or lose expression of the targeted antigen, thereby escaping immune surveillance. This phenomenon, sometimes referred to as “antigen escape”, is further complicated by clonal heterogeneity, where some tumor subpopulations may already harbor reduced antigen levels. Consequently, therapies that rely on stable antigen expression may become less effective over time, necessitating additional or alternative targets to maintain durable immune control. Moreover, some degree of normal tissue expression of these antigens cannot be completely ruled out, underscoring safety considerations when deploying potent immune effectors [40]. Current data directly comparing antigen downregulation rates between prostate cancer and other cancers undergoing antigen-directed therapies are limited. Antigen downregulation, including the reduced expression of targets like PSMA, PSCA, or MHC class I molecules, is a significant resistance mechanism in prostate cancer [41]. Similar phenomena occur in other malignancies; for instance, CD70-targeted therapies show varying response rates, with approximately 70% efficacy in T-cell lymphoma but only 8% in renal cell carcinoma, reflecting differences in antigen expression and the tumor microenvironment [42]. Systematic comparative studies across different cancers are needed to enhance understanding of resistance mechanisms and optimize therapeutic strategies.

The presence of PSAs in an otherwise low-mutation cancer highlights the critical role of combination regimens. Checkpoint inhibitors, metabolic modulators, and novel immuno-engineering strategies (such as CAR T cells and T-cell receptor-engineered T cells) have the potential to reinforce antigen-driven therapies by reinvigorating T cells and counteracting immunosuppressive effects. However, balancing efficacy and safety remains a crucial challenge, particularly given the risks of normal tissue toxicity and antigen loss [39].

Thus, the immunological landscape of prostate cancer presents a formidable challenge owing to its low immunogenicity, extensive immunosuppression, and intricate AR-driven biology. Although recent insights have clarified key evasion mechanisms, ranging from checkpoint upregulation to metabolic constraints, translating these findings into meaningful clinical benefits has been difficult. Future research should prioritize methodological rigor, encompassing larger biomarker-driven trials that account for both tumor-intrinsic factors and patient-specific variables. A core element of success involves merging rational immunotherapies with established treatments such as ADT, second-generation AR inhibitors, or evolving targeted agents. Additionally, harnessing advances in T-cell engineering, genome editing, and computational immuno-oncology could help decode prostate cancer’s immune resistance. By integrating historical perspectives, present evidence, and emerging possibilities, we can refine immunotherapeutic strategies and ultimately offer a more durable control of a disease long considered immunologically intractable [38,39].

## 3. Past and Present of Immunotherapy Approaches for Prostate Cancer

### 3.1. Early Attempts and Key Lessons

Immunotherapeutic research in prostate cancer has roots stretching back to at least the 1980s, when rudimentary vaccines and tumor lysate–based strategies were developed to trigger antitumor immunity. Although these initial trials produced minimal clinical benefits, partly because of small sample sizes, heterogeneous patient populations, and limited immune-monitoring tools, they provided foundational insights. Specifically, they highlighted the importance of robust antigen presentation, the potential need for immunostimulatory adjuvants, and the capacity of the tumor for immune evasion [43,44].

Dendritic cell (DC) vaccines were developed in the 1990s using patient-derived DCs pulsed with tumor antigens to induce T-cell responses. Early phase data suggested enhanced immunogenicity but only modest clinical improvements, presumably because of insufficient infiltration or function of tumor-specific T cells. Nevertheless, these collective findings set the stage for the later development and U.S. Food and Drug Administration approval of sipuleucel-T, which demonstrated that prostate tumors are not categorically immune-inert [43,44,45].

Critically, many early studies lacked rigorous mechanistic correlative endpoints, making it difficult to discern which immunological variables—T-cell repertoire diversity, Treg frequency, MDSC levels, etc.—were most predictive of outcomes. Modern trials now incorporate more sophisticated immunophenotyping and genomic profiling techniques, reflecting a more nuanced appreciation of the tumor immune landscape [38,44].

### 3.2. Current Strategies and Clinical Experience: Immunotherapeutic Approaches

**Figure 3:**
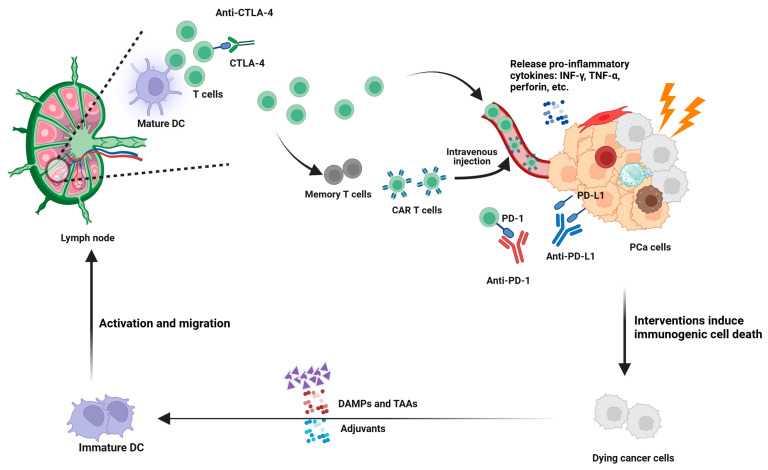
Mechanisms of immune activation and targeted strategies in prostate cancer immunotherapy. Interventions (e.g., chemotherapy, radiotherapy, or oncolytic viruses) can induce immunogenic cell death in tumor cells, releasing damage-associated molecular patterns (DAMPs) and tumor-associated antigens (TAAs). These signals, often aided by adjuvants, activate immature dendritic cells (DCs), prompting their maturation and migration to regional lymph nodes. There, mature DCs present TAAs to T cells and help initiate robust antitumor responses, which are further amplified by checkpoint-blocking antibodies (e.g., anti-CTLA-4). The expanded population of tumor-specific T cells, including memory T cells, can be infused as chimeric antigen receptor (CAR) T cells or continue to circulate, infiltrating the prostate cancer (PCa) lesion. Once at the tumor site, T cells produce proinflammatory cytokines such as IFN-γ, TNF-α, and perforin, leading to cancer cell lysis. Meanwhile, therapies targeting the PD-1/PD-L1 axis counteract tumor-induced immunosuppression, augmenting T-cell cytotoxicity. Collectively, these strategies harness immune activation at multiple stages—antigen release, DC-mediated priming, T-cell expansion, and effector function—to optimize the eradication of prostate cancer cells. Created with BioRender.com.

Multiple immunotherapeutic strategies have been explored in prostate cancer, each aiming to overcome different facets of the tumor’s immunosuppressive microenvironment. Figure 3 provides an overview of the major immunotherapy strategies applied to prostate cancer, comprehensively presenting the mechanism of each approach and its role within the TME. In the following subsections, we will discuss in detail how these strategies have been developed, tested, and combined in clinical settings.

#### 3.2.1. Immune Checkpoint Blockade

ICIs directed against CTLA-4 (ipilimumab), PD-1 (pembrolizumab and nivolumab), or PD-L1 have transformed the treatment landscape of tumors, such as melanoma and non–small cell lung cancer, where tumor-infiltrating lymphocytes are abundant. By contrast, prostate cancer’s “cold” immune profile has limited the efficacy of single-agent checkpoint blockade, with early monotherapy trials often reporting response rates under 10–15% [38,46]. Although their effect is modest in unselected prostate cancer populations, these inhibitors have shown more promising results in subsets characterized by dMMR or MSI-H. A few studies—often small or single-arm and generally providing level II evidence (which refers to data derived from non-randomized controlled trials or well-designed observational studies, as opposed to level I evidence from high-quality randomized controlled trials and meta-analyses and level III evidence from case series, uncontrolled studies, or expert opinion)—have indicated that PD-1 inhibitors may generate higher response rates in these biomarker-defined groups [38,47]. Trials combining ipilimumab with radiotherapy or PD-1 inhibition have demonstrated incremental improvements in progression-free survival, although the advantages in overall survival remain elusive. The low TMB and highly immunosuppressive microenvironment of prostate cancer likely dampen checkpoint inhibitor activity [38]. Some studies suggest that early intervention with ICIs during the biochemical recurrence (BCR) of prostate cancer may be more effective. However, conclusive evidence supporting this approach is still limited. Some early stage studies have reported significant PSA reductions in BCR patients using vaccine-based immunotherapies, but these findings are based on small-scale and exploratory research, making it difficult to draw definitive conclusions [38,40,48]. Some data suggest better responses when the disease burden is relatively low or when treatment is introduced at earlier disease stages, such as during biochemical recurrence; however, this observation requires validation in larger trials (level III evidence). Ongoing research continues to explore expanded combinations with poly-ADP ribose polymerase (PARP) or TGF-β inhibitors, alongside artificial intelligence (AI)-driven biomarker strategies aimed at refining patient selection and enhancing therapeutic efficacy [38,49]. In this context, the combination of atezolizumab and androgen receptor pathway inhibitors (ARPIs, agents that block androgen receptor signaling to suppress tumor growth) in mCRPC has been evaluated with mixed results. The phase 3 CONTACT-02 trial compared cabozantinib plus atezolizumab to a second novel hormonal ARPI in patients whose disease had progressed on one prior ARPI, demonstrating a statistically significant improvement in progression-free survival (PFS) with the combination (6.3 months vs. 4.2 months) but no statistically significant overall survival (OS) benefit in the intention-to-treat population. A subgroup benefit was noted in individuals with liver or bone metastases, and although there was a trend toward better OS (16.7 months vs. 14.6 months), it was not statistically significant. Treatment-related adverse events were more frequent with cabozantinib plus atezolizumab [50]. Another phase 3 study, IMbassador250, examined atezolizumab plus enzalutamide versus enzalutamide alone in men previously treated with abiraterone, finding longer PFS in tumors exhibiting high PD-L1 IC2/3, CD8 expression, and specific immune gene signatures [51]. Overall, immunotherapy has produced disappointing results in unselected mCRPC populations, highlighting the importance of cautiously interpretating the CONTACT-02 findings. Nonetheless, these data suggest that cabozantinib plus atezolizumab may represent a potential treatment option for certain patients progressing on first-line ARPI, particularly if future research clarifies how to best identify and manage those most likely to benefit.

#### 3.2.2. Induction of ICD

The concept of ICD arose from the finding that certain therapeutic interventions, notably specific chemotherapeutics or radiotherapy, not only destroy tumor cells but also stimulate an immune response against released antigens. This process involves DAMPs such as calreticulin, HMGB1, and extracellular ATP, which help activate and recruit antigen-presenting cells [52]. In prostate cancer, in which immunosuppression is prominent, ICD is viewed as a potential mechanism for shifting the tumor milieu into one that is more amenable to T-cell infiltration and recognition. Clinical studies have primarily focused on cytotoxic agents, such as docetaxel or cabazitaxel, as well as radiation, sometimes administered with immunotherapies [53]. Although preclinical models support the synergy of ICD inducers with vaccines or checkpoint inhibitors, the clinical data have been inconsistent [54,55]. Some phase II trials combining docetaxel with immunotherapy have shown improved immunological readouts, although the definitive survival gains remain unclear. Most evidence has been derived from exploratory endpoints rather than large-scale randomized comparisons, suggesting the need for rigorous biomarker validation (level II/III evidence). Methodological hurdles include identifying uniform ICD markers in patient samples and accounting for tumor heterogeneity, especially in patients with bone metastases [53,56]. Future studies will aim to refine dosing schedules, explore novel ICD-promoting agents, and pair them with checkpoint blockade- or myeloid-directed therapies. By converting the normally silent death of tumor cells into a robust immunogenic event, these strategies can overcome the immune inertia of prostate cancer and catalyze sustained T-cell–mediated tumor control [53,57].

#### 3.2.3. TME Reversal (Immunologically “Hot” Conversion)

Prostate cancer’s microenvironment is characterized by dense immunosuppressive elements, including elevated TGF-β, IL-10, and M2-like macrophages. This “cold” milieu contributes to poor T-cell infiltration and suboptimal immunotherapy responses [57,58]. TME reversal strategies encompass interventions designed to spark a local proinflammatory response, thereby enabling an effective immune response. Oncolytic viruses and low-dose radiotherapy are thought to expose tumor antigens and induce localized inflammatory signals that improve T-cell trafficking [2]. Other approaches involve inhibiting CSF-1R to reprogram M2 macrophages or blocking IDO to alleviate metabolic barriers [59,60]. As prostate tumors typically rely on multiple overlapping inhibitory pathways, single-agent TME modulators seldom produce durable clinical outcomes [61]. Combining TME-targeted agents with established regimens, such as chemotherapy, radiotherapy, or targeted inhibitors, may temporarily open a “window of opportunity” for enhanced T-cell-mediated killing [62]. However, patient heterogeneity (metastatic profiles and baseline immune states) complicates the interpretation of outcomes, and standardized methods for measuring changes in TME composition are not yet well established [63].

#### 3.2.4. Tumor Vaccines

Vaccines directed against prostate cancer antigens, including PSA, PAP, and PSMA, activate antigen-specific T-cell responses [64]. The landmark approval of sipuleucel-T in 2010 substantiated the viability of prostate cancer immunotherapy, providing level I evidence of a survival benefit in metastatic CRPC despite minimal changes in conventional endpoints such as PSA or imaging responses [65]. Subsequent vaccine platforms, from peptide- and viral vector-based constructs to emerging mRNA formulations, have yielded encouraging immunogenicity data, manifested as T-cell proliferation or interferon-gamma release, but have not consistently produced clear improvements in overall or progression-free survival [66]. The disconnect between immunological readouts and clinical endpoints may reflect the delayed nature of immune activation as well as the tumor’s ability to deploy compensatory escape mechanisms [67]. Retrospective analyses have suggested that patients with a lower disease burden or early stage metastatic disease might derive more pronounced benefits; however, a unified biomarker to guide vaccine use remains elusive. Current research focuses on personalized vaccine approaches, including multi-epitope or multivalent designs, integration with checkpoint inhibition, and advanced antigen discovery platforms that incorporate AI to identify patient-specific neoantigens [68].

#### 3.2.5. Bispecific T-Cell Engagement

Xaluritamig (AMG 509) is a bispecific T-cell engager (BiTE) immunotherapy under development for mCRPC. It binds to the six-transmembrane epithelial antigen of the prostate (STEAP1), which is overexpressed in most prostate cancers and shows limited expression in normal tissues. STEAP1 is considered a promising therapeutic target, partly due to its potentially more homogeneous expression compared to PSMA. Xaluritamig contains two STEAP1-binding domains and a CD3-binding domain, enabling the recognition of both cancer cells and T cells and leading to efficient T-cell-mediated cytotoxicity against STEAP1-expressing cells [69]. In a phase 1 clinical trial involving heavily pretreated mCRPC patients, Xaluritamig was administered weekly by intravenous infusion, with a maximum tolerated dose (MTD) of 1.5 mg. Common treatment-related adverse events included cytokine release syndrome (CRS), fatigue, and myalgia, with most CRS reported as grade 1 or 2. Regarding efficacy, PSA declines were observed in 67 patients, 16 of whom experienced a partial response. A PSA50—defined as a reduction of at least 50% in serum prostate-specific antigen (PSA) levels from baseline—response was seen in 49% of patients, while 30% achieved a PSA90 response, defined as a reduction of at least 90% in serum PSA levels from baseline, and the overall response rate (ORR) was 24%. Notably, at doses of 0.75 mg or higher, the ORR was 41%, PSA50 response reached 59%, and PSA90 response was 45.1%. Adverse events of grade 3 or higher were reported in 66% of patients, with 9% experiencing grade 4 events and 16% discontinuing treatment due to adverse events [70,71]. Amgen is currently advancing Xaluritamig to a phase 3 trial in the second-line mCRPC setting, where the management of CRS and musculoskeletal side effects remains a key consideration [71]. BiTE therapies hold significant promise for treating prostate cancer, particularly mCRPC, but they also raise several safety concerns that need to be carefully addressed. One major issue is the on-target, off-tumor effect: BiTEs target tumor-associated antigens that can also be expressed in normal tissues, potentially leading to unintended immune attacks on healthy cells—for example, PSMA-targeting BiTEs may affect normal tissues that express PSMA [72]. In addition, these therapies can induce inflammatory adverse events and immune effector cell-related toxicities, such as cytokine release syndrome, immune effector cell-associated neurotoxicity syndrome, and other inflammatory complications like myocarditis, vasculitis, or pneumonitis, with patients who have pre-existing inflammatory conditions being at higher risk [73]. Continuous T-cell activation by BiTEs can also lead to T-cell exhaustion, impairing their long-term functionality and limiting the durability of therapeutic responses. Moreover, the induction of high levels of anti-drug antibodies can reduce the efficacy of BiTE therapies and promote drug resistance, as observed in early clinical trials. Finally, dose-limiting toxicities—especially high-grade (grade 3/4) treatment-related adverse events—further complicate dose management and treatment continuation [72]. Addressing these challenges will require precise patient selection, the development of combination therapies, and innovative BiTE design strategies, along with ongoing clinical research to optimize the safety and efficacy of these treatments.

#### 3.2.6. Immune Adjuvants

Immune adjuvants bolster the activity and longevity of the antitumor immune response [74]. Traditional examples include granulocyte macrophage colony-stimulating factor (GM-CSF), TLR agonists, and STING agonists, which promote DC maturation and amplify inflammatory signaling [74]. Sipuleucel-T leverages GM-CSF to activate patient-derived antigen-presenting cells, thereby illustrating the practical utility of adjuvants in prostate cancer [65]. Although single-agent therapy has typically yielded modest results, other agents such as CD40 agonists are being investigated [75]. Combinatorial strategies that integrate adjuvants with radiotherapy or checkpoint inhibitors have shown moderate improvements in immunological outcomes; however, confirmation through larger randomized trials is necessary [76]. STING agonists, which may induce robust type I interferon responses, face challenges in drug delivery and toxicity management [74]. The complexity of the immune landscape in prostate cancer suggests that adjuvants alone rarely reverse deep-seated tolerance mechanisms, driving a broader consensus that they should be used alongside vaccines, TME modulators, or checkpoint blockades [67]. The further development of nanoparticle- or liposome-based delivery systems could optimize tumor targeting while sparing healthy tissues [77].

#### 3.2.7. CAR T-Cell Therapy

CAR T cells, genetically engineered to recognize tumor-specific antigens, have revolutionized the treatment of certain hematological cancers. However, replicating these successes in prostate cancer poses substantial challenges. Several phase I/II trials have targeted surface antigens such as PSMA or PAP. Although some partial responses have been observed, large-scale data confirming their clinical efficacy remain limited [68]. The immunosuppressive prostate TME, replete with TGF-β and IL-10, quickly impairs the proliferation and function of infused CAR T cells [67]. There is also a risk of on-target, off-tumor toxicity if these antigens appear in normal tissues. Next-generation “armored” CAR T-cell constructs aim to overcome these obstacles by incorporating cytokine release elements or engineering receptors that resist inhibitory signals. Clustered regularly interspaced short palindromic repeat (CRISPR)-based gene editing has opened up the possibility of removing checkpoint molecules or creating multi-specific CAR designs. Although these concepts are promising, scaling up production, controlling costs, and managing potential toxicities remain significant hurdles and further validation in larger studies is essential [68].

#### 3.2.8. Interventions Targeting Barriers to TME Infiltration

Prostate tumors often develop within the fibrotic stroma and can metastasize to the bone, where structural and biochemical barriers impede immune cells or drug penetration. Abnormal tumor vasculature, dense extracellular matrix components, and limited lymphatic networks further constrain cell infiltration and delivery. Strategies to address these barriers include antifibrotic approaches such as TGF-β or LOX inhibition, enzymatic breakdown of the extracellular matrix (e.g., collagenase), and vascular normalization using targeted agents or radiotherapy. Although preclinical and small clinical studies suggest that these methods may improve T-cell access, personalizing their use is challenging because each patient’s tumor stroma differs in composition and remodeling potential. Overly aggressive stromal disruption can harm normal tissues, highlighting the need for precise biomarker-based guidance. Robust multi-institutional trials are necessary to define the optimal balance between alleviating physical barriers and preserving healthy structural integrity [67]. At the same time, targeting stromal barriers in conjunction with immunosuppressive networks in prostate cancer remains a formidable challenge. Recent experimental and clinical data have shown promising approaches in this regard. For example, antiangiogenic therapy—exemplified by the combination of cabozantinib with atezolizumab in a phase Ib trial—has demonstrated improved vascular regulation and increased T-cell infiltration in mCRPC patients, with an objective response rate of 32% [2]. Additionally, the local administration of immunotherapeutic agents has been explored as a means to bypass stromal barriers [78]. In addressing immunosuppressive networks, STAT3 inhibition via CpG-STAT3 siRNA has shown potential in preclinical models, particularly in overcoming immunosuppression mediated by granulocytic myeloid-derived suppressor cells (G-MDSCs) [79]. Combination immunotherapy, such as that evaluated in the CheckMate 650 trial combining nivolumab and ipilimumab, has also demonstrated clinical activity in mCRPC patients [27]. Further, strategies targeting immunosuppressive cells—like the use of low-dose metronomic cyclophosphamide to deplete regulatory T cells—and emerging research identifying club-like epithelial cells as potential drivers of immunosuppression, offer new avenues for intervention [2]. While many of these approaches are still in experimental stages or have shown limited efficacy in clinical trials, the complex nature of prostate cancer’s immunosuppressive environment suggests that combination strategies targeting multiple pathways may be necessary to achieve significant clinical benefit. Ongoing research continues to explore novel approaches to overcome these barriers and enhance the overall efficacy of immunotherapy in prostate cancer.

#### 3.2.9. Clinical Trials of Immunotherapeutic Approaches: Current Evidence and Challenges

Multiple clinical trials—both active and completed—have explored diverse immunotherapeutic strategies in prostate cancer. For checkpoint blockade, the CheckMate 650 trial (phase II, NCT02985957) combined nivolumab and ipilimumab in metastatic castration-resistant prostate cancer (mCRPC), demonstrating modest efficacy in biomarker-selected subgroups [80], whereas the KEYNOTE-199 study (phase II, NCT02787005) tested pembrolizumab monotherapy but showed limited response rates in unselected patients [81]. The recently reported CONTACT-02 trial (phase III, NCT04446117) assessed cabozantinib plus atezolizumab versus a second-line ARPI in mCRPC patients who had progressed on one prior novel hormonal agent, achieving improved progression-free survival but no overall survival benefit in the intention-to-treat population [82]. In the vaccine arena, the IMPACT trial (phase III, NCT00065442) established sipuleucel-T (targeting prostatic acid phosphatase) as a viable immunotherapy by demonstrating a survival benefit [4], while the PROSPECT trial (phase III, NCT01322490) evaluated the viral vector-based PROSTVAC vaccine and did not meet its primary survival endpoint [83]. To induce immunogenic cell death (ICD), smaller phase I/II studies (e.g., NCT02649855) have combined docetaxel or cabazitaxel with vaccines or checkpoint inhibitors, yielding encouraging immunologic readouts but inconclusive survival outcomes [84]. TME reversal has been pursued through IDO inhibitors (e.g., epacadostat in NCT03374488) and CSF-1R inhibitors (NCT01004861), albeit with limited single-agent efficacy [4]. Bispecific T-cell engagers (BiTEs) such as Xaluritamig (AMG 509) are under active investigation in heavily pretreated mCRPC (phase I, NCT04221542), showing promising PSA responses but significant rates of cytokine release syndrome [69]. Investigations into chimeric antigen receptor (CAR) T cells, predominantly targeting prostate-specific membrane antigen (PSMA) via multiple phase I/II trials (NCT03089203, NCT03356795), have reported occasional partial responses but face challenges from on-target/off-tumor toxicity and the immunosuppressive tumor microenvironment [85]. Lastly, various approaches aiming to overcome physical barriers to T-cell infiltration include trials of TGF-β inhibition (NCT02423343) and antiangiogenic agents (cabozantinib + atezolizumab in COSMIC-021, NCT03170960), some of which have shown transient benefits in clinical endpoints and remain under active investigation in larger, randomized studies [4]. A comprehensive summary of major immunotherapeutic strategies and associated key clinical trials in prostate cancer is provided in Table 2, highlighting various approaches, representative agents, and key clinical findings along with their respective challenges.

#### 3.2.10. Overall Critique and Future Direction

Historically, single-agent immunotherapies for prostate cancer have yielded limited results, highlighting the importance of multimodal strategies. Multiple immunotherapeutic approaches have been explored in prostate cancer, ranging from ICIsto CAR T-cell therapy (Table 3) [68]. Although single-agent strategies often yield limited responses, rational combination regimens show promise in overcoming the immunosuppressive TME. Extensive immunosuppressive networks in this malignancy often require simultaneous or sequential interventions, such as coupling ICD inducers with checkpoint inhibitors or combining TME reversal approaches with targeted radiotherapy [67]. Precision oncology initiatives, including genomic profiling for dMMR, MSI-H, and DNA repair defects (e.g., BRCA2), promise to refine patient selection and improve therapeutic outcomes by focusing on those most likely to benefit from immunotherapy. Clinical trial designs are also adapted through the use of adaptive or basket trials that incorporate real-time biomarker assessments, although the lack of standardized immunological and molecular endpoints can hinder data interpretation [68]. Technological convergence in areas such as next-generation CAR T cells, mRNA vaccine platforms, and advanced data analytics offers new avenues for intensifying immune responses [86]. However, the manufacturing complexities, economic constraints, and long-term safety considerations must be carefully managed as these approaches evolve [68]. The established survival benefit of sipuleucel-T confirms that prostate cancer is not insurmountably “cold”, suggesting that further refinement in combination strategies, timing, and biomarker-guided care can harness the power of the immune system to better control or even transform the disease course [65].

## 4. Predictive Biomarkers and Patient Stratification

Efforts to refine patient selection for immunotherapy in prostate cancer have drawn on decades of translational research aimed at linking molecular features to clinical outcomes [77]. Historically, immunohistochemical assessments of PD-L1 expression, which is influential in other solid tumors, have proven to have limited prognostic value in prostate cancer [67]. This has prompted a shift toward mechanistic markers that reflect the inherently low immunogenicity of prostate cancer, such as mismatch repair deficiency (dMMR), a microsatellite instability-high (MSI-H) phenotype, and CDK12 gene alterations [87]. Notably, MSI-H/dMMR status has emerged as the most validated predictive biomarker in this setting, facilitating the FDA approval of pembrolizumab for MSI-H/dMMR solid tumors [88]. However, not all patients with these molecular features respond favorably, underscoring the need for additional biomarkers [47].

Tumor mutational burden (TMB) has also gained attention as a potential predictor, with higher TMB often correlating with improved responses in other cancer types, though its prognostic utility in prostate cancer remains under investigation [89]. In contrast, PD-L1 expression—which can predict response in melanoma, lung cancer, and other tumors—has produced inconsistent results in prostate cancer [90]. Emerging biomarkers include homologous recombination deficiency (HRD), CDK12 inactivation, and elevated levels of soluble PD-L1 (sPD-L1), which have been associated with shorter progression-free survival [91,92]. These findings highlight how the molecular biology of prostate cancer diverges from more immunogenic malignancies and emphasize the need for ongoing research to validate and refine the use of these biomarkers.

### 4.1. Biomarkers for Immunotherapy Response

MSI-H and dMMR were among the earliest recognized predictors of immunotherapeutic benefit, reflecting an elevated mutational load capable of eliciting stronger T-cell responses. Despite rigorous preclinical and clinical investigations (level II evidence from basket trials and retrospective analyses), MSI-H/dMMR status is relatively rare in prostate cancer—occurring in only approximately 2–3% of cases—which constrains its utility in widespread practice [93,94]. Nevertheless, patients with MSI-H/dMMR prostate tumors exhibit promising immunotherapy response rates ranging from 25% to 60%, with some studies reporting PSA declines of ≥50% in about 50–54.5% of cases [93,94]. These response rates are comparable to, or even slightly higher than, those observed in MSI-H/dMMR tumors in other malignancies (ranging from 34% to 69%) [95]. Meanwhile, TMB offers another perspective; although a higher TMB theoretically broadens the neoantigen repertoire, truly high TMB values remain the exception rather than the rule in prostate cancer [77]. Importantly, emerging data from prospective trials suggest that certain hypermutated subsets may respond more favorably to checkpoint blockade [67]. Given these favorable outcomes, it is crucial to identify MSI-H/dMMR patients—especially those with advanced disease—for tailored immunotherapeutic interventions.

Gene fusions, such as CDK12, also stand out for their associated with hypermutation and putative immunogenicity. While this hypothesis is supported by pilot studies, validating CDK12 status as a definitive predictive biomarker requires larger, methodologically rigorous trials (level I–II evidence pending). Building on this, it is important to recognize that DNA repair gene mutations—such as BRCA2—also play a critical role in influencing treatment responses and guiding personalized therapeutic strategies in prostate cancer. The TALAPRO-2 trial and related studies demonstrate the importance of genetic and other biomarkers in guiding prostate cancer treatment choices. For example, BRCA1/2 and ATM mutations predict response to ARPIs and PARP inhibitors, with the TALAPRO-2 study showing that the combination of talazoparib (TALA) and enzalutamide (ENZA) was more effective in patients harboring these mutations. Similarly, TMPRSS2-ERG and RB1 alterations emerged as candidate biomarkers for predicting response to TALA + ENZA treatment, as these alterations were associated with superior efficacy compared to placebo plus ENZA. In addition to these genetic markers, other biomarkers also play significant roles in treatment response prediction. PTEN loss, for instance, not only protects p53 but also increases cancer cell sensitivity to chemotherapy, and the concurrent loss of PTEN and TP53 is linked to a more aggressive prostate cancer phenotype. Moreover, TP53 mutations have been shown to predict response to 177Lu-PSMA radioligand therapy and may also be useful in forecasting neoadjuvant chemotherapy outcomes in other cancers [96].

Liquid biopsy techniques, including circulating tumor DNA (ctDNA) and circulating tumor cells, hold promise for real-time insights into tumor evolution. Theoretically, these assays can guide immunotherapy timing or modifications by detecting resistance mutations or shifts in molecular profiles. Both the PSMAfore and TheraP studies highlight the significance of ctDNA in mCRPC patients, demonstrating that lower ctDNA fractions are associated with better responses to 177Lu-PSMA-617 therapy and suggesting ctDNA as a potential biomarker for treatment selection and prognosis in mCRPC [97,98]. However, standardization remains a challenge, as the assay sensitivity and clinical significance of minor variant alleles vary widely across platforms [77].

### 4.2. Prostate Cancer-Specific Markers

PSA remains firmly entrenched in clinical practice as a diagnostic and monitoring tool [65]. However, its predictive value for immunotherapeutic responses is less consistent. Numerous reports have documented cases in which PSA levels do not decline appreciably despite clear clinical or radiographic improvements with therapies such as sipuleucel-T or checkpoint inhibitors. This disconnection underscores the need for additional markers, potentially PSA kinetics, combined with immune-related imaging or next-generation sequencing data, to capture the full spectrum of antitumor effects [99].

Advances in PSMA-based imaging, exemplified by PSMA-positron emission tomography, have revolutionized the detection of metastatic diseases. Beyond mere localization, these techniques might soon support “precision interventions” such as targeted radioligand therapy, which can be combined with immunotherapy for synergistic effects [100]. Other prostate-related antigens, such as PAP and STEAP1, are under investigation as vaccines or CAR T-cell targets, although robust evidence for their role in guiding patient stratification remains scarce [101]. Consequently, the field is gravitating toward multi-omics, a strategy that integrates information on tumor-specific antigens, immune cell infiltration, and genomic aberrations to construct biomarker panels reflecting both tumor biology and immune context [102].

As summarized in Table 4, established and emerging biomarkers—such as MSI-H/dMMR status, CDK12 alterations, and tumor mutational burden—hold promise for guiding immunotherapy in prostate cancer. However, each biomarker presents unique challenges related to assay standardization and prevalence [103].

### 4.3. Precision Medicine and Comprehensive Profiling

The future of biomarker discovery in prostate cancer increasingly favors a comprehensive approach integrating the genome, transcriptome, proteome, and metabolome. By capturing the complexity of tumor–immune system interactions, such profiling holds the potential to identify novel therapeutic targets and refine the eligibility criteria for immunotherapy trials. This precision oncology framework has already generated success stories in other malignancies, particularly in breast cancer (BRCA-mutated tumors) managed with PARP inhibitors, and is a gradually reshaping treatment algorithms for a subset of prostate cancers [106]. In this context, recent findings from the SPARTAN phase III trial further underscore the value of advanced digital pathology techniques. The trial demonstrated that digital pathology-based multimodal artificial intelligence (MMAI), integrating digitized H&E slides and clinical parameters (Gleason score, age, T stage, PSA), effectively predicts prognosis and treatment response in men with nmCRPC. The MMAI model outperformed traditional clinical risk stratification by identifying high-risk patients with significantly shorter metastasis-free survival (MFS) and second progression-free survival (PFS2). Notably, high-risk MMAI patients receiving apalutamide had substantially improved MFS compared to those receiving a placebo (HR 0.19, *p* < 0.005), with a significant interaction suggesting these patients may derive greater treatment benefit. This first-ever application of MMAI in CRPC highlights its potential for personalized treatment strategies, though further validation is needed [107].

Despite the considerable momentum, challenges remain. Adaptive trial designs, which allow for real-time modifications based on evolving biomarker data, are resource-intensive and are typically feasible only at specialized centers. Nevertheless, as next-generation sequencing becomes more cost-effective and bioinformatics workflows become more robust, the integration of multi-omic analyses into routine clinical decision-making may accelerate. Such initiatives could also mitigate the risk of exposing patients to therapies that are unlikely to confer benefits while maximizing the yield of combination regimens tailored to individual molecular landscapes [108].

## 5. Combination Therapies

The concept that prostate cancer’s low immunogenicity can be overcome via rationally designed combination approaches has emerged from early smaller-scale immunological assessments. Since then, numerous trials have attempted to synergize immune-based strategies with androgen deprivation, chemotherapy, targeted agents, and radiotherapy. Despite mixed or even conflicting outcomes, this area remains a central focus of clinical research, underscoring the complexity of modulating the TME in diseases characterized by heterogeneous biology and variable host immunity [109]. Several combination strategies have been investigated to enhance the efficacy of immunotherapy by targeting diverse aspects of prostate cancer’s immunosuppressive landscape. A summary of these strategies, underlying mechanisms, representative clinical trials, key outcomes, and associated challenges is provided in Table 5.

### 5.1. Hormonal Therapy Plus Immunotherapy

Historically, ADT has been the mainstay of treatment for advanced prostate cancer, reflecting pioneering work in the mid-20th century that linked hormonal manipulation to tumor regression. More recently, preclinical models and pilot trials (level II evidence) have indicated that short-term ADT modulates the immune milieu, most notably by reducing certain immunosuppressive myeloid subsets and temporarily enhancing T-cell infiltration. However, attempts to parlay these shifts into meaningful survival gains with concurrent checkpoint inhibition or therapeutic vaccines have yielded inconsistent results, reflecting the complex interplay among timing, sequencing, and interpatient variability in immune competence.

One critical question centers on the optimal timing for introducing immunotherapy: Should it be deployed concurrently with ADT initiation in the setting of minimal residual disease or later, once castration resistance emerges? Variations in trial design, hormone regimens (e.g., luteinizing hormone-releasing hormone agonists vs. antagonists), and endpoints (radiographic progression vs. overall survival) make direct comparison difficult. Developing a more unified approach, standardizing eligibility criteria, and incorporating immunologic biomarkers could help disentangle these conflicting findings [39,110].

### 5.2. Chemotherapy, Targeted Therapies, and Beyond

Chemotherapy, particularly docetaxel or cabazitaxel, remains the cornerstone of care for CRPC [116]. Multiple studies have suggested that certain cytotoxic agents can trigger ICD, creating opportunities to improve antigen presentation and T-cell activation [111]. However, the durability of these effects and their net clinical effect vary, with some trials reporting improvements in T-cell density and others finding little synergy when chemotherapy is combined with checkpoint blockade [112].

Targeted therapies, particularly PARP inhibitors, represent the second major pillar of combination efforts. By exploiting the DNA repair deficits in a subset of tumors (e.g., those bearing BRCA2 mutations), PARP inhibitors may enhance immunogenicity in this population. Although preliminary signals of efficacy have emerged, questions remain as to whether these benefits extend to broader patient cohorts lacking clearly defined DNA repair anomalies [104]. In parallel, a host of novel agents aimed at blocking immunosuppressive pathways—spanning from TGF-β antagonists to MDSC and TAM inhibitors—are under exploration [117]. The principal challenge remaining is to determine how to sequence or layer these drugs without exacerbating toxicity or inadvertently crippling the immune function.

### 5.3. Radiotherapy and Immunotherapy

Radiotherapy’s capacity to provoke systemic immune responses—popularly referenced as the “abscopal effect”—has long captured scientific curiosity but eluded consistent clinical demonstration [113]. In prostate cancer, stereotactic body radiotherapy and PSMA-targeted radioligand therapy (e.g., 177Lu-PSMA-617) are prime candidates for eliciting ICD, potentially amplifying the efficacy of checkpoint blockade or vaccine-based strategies [118]. Early phase studies documented sporadic abscopal responses, prompting optimism that refinement of the radiation dose, fractionation, and timing of concurrent immunotherapy might yield reproducible synergy [114]. The PRESERVE-006 study (NCT05682443) is a groundbreaking phase 1/2 clinical trial investigating the combination of Pluvicto (177Lu-PSMA therapy) with BNT316/ONC-392 (an anti-CTLA-4 antibody) in mCRPC patients who have progressed after ARPI treatment. This study, designed with a 2:1 randomization between the combination therapy and Pluvicto alone, aims to evaluate whether the synergy of immunotherapy and targeted radiotherapy can improve outcomes in mCRPC. With rPFS as the primary endpoint, this trial has the potential to reshape treatment strategies for advanced prostate cancer, offering new hope for patients with limited options by exploring the efficacy of combining targeted radiation delivery to PSMA-expressing prostate cancer cells with immune-boosting effects [119]. However, methodological disparities and variations in the intrinsic radiation sensitivity of tumors create uneven outcomes. Future trial designs guided by real-time immune profiling could help identify the subgroups most likely to benefit from combined radiotherapy and immunotherapy regimens [58]. Table 4 summarizes several combination regimens, ranging from hormonal therapy plus immunotherapy to multi-target approaches, that have been investigated to overcome prostate cancer’s immunosuppressive landscape [104].

## 6. Limitations and Future Directions

Despite incremental gains over the past decade, immunotherapy for prostate cancer delivers only modest response rates in most unselected populations [5]. Coupled with safety issues surrounding immune-related adverse events (irAEs), this underscores the reality that a single-agent checkpoint blockade is frequently inadequate for tumors that are inherently replete with immunosuppressive mechanisms [120]. Recognizing this, researchers have increasingly turned to combination therapies, ranging from dual checkpoint inhibitors to quadruple regimens involving radiotherapy and hormonal manipulation. However, mounting concerns about cumulative toxicity, cost, and trial complexity warrant careful consideration [115].

### 6.1. Novel Technologies and Therapeutic Concepts

Next-generation CAR T cells designed to withstand TGF-β-rich environments or secrete immunostimulatory cytokines offer a cutting-edge solution to prostate cancer’s hostile microenvironment, although robust evidence of clinical efficacy remains limited to early stage trials [121]. Similarly, CRISPR-based gene editing could enable the precise removal of inhibitory receptors or the incorporation of enhanced effector functions into T or natural killer cells. This offers potential for durable, self-perpetuating immune control; however, regulators and clinicians remain vigilant about the off-target effects and long-term consequences of genetically modifying immune cells [122].

Messenger RNA (mRNA) vaccine platforms, propelled to prominence by recent successes in infectious diseases, offer an appealing avenue for rapidly generating patient-specific antigens. Early prostate cancer studies indicated a capacity to stimulate antigen-specific T cells; however, whether these responses translate into survival benefits awaits confirmation in more expansive, randomized settings [123]. Finally, AI-driven analyses are emerging to integrate molecular, imaging, and clinical data, potentially clarifying subtle patterns that predict immunotherapy responsiveness. Although AI approaches hold great promise, particularly in complex cancers such as prostate cancer, concerns about data heterogeneity and algorithm transparency persist [124].

### 6.2. Precision Oncology Approaches

**Figure 4:**
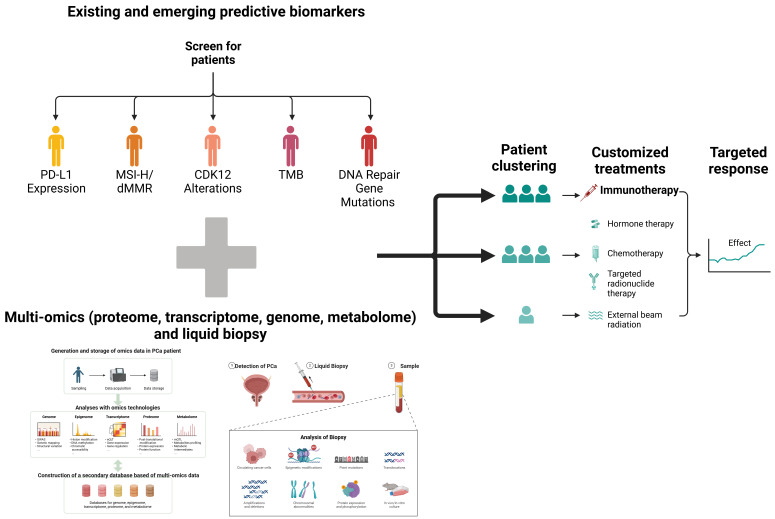
Predictive biomarkers and multi-omics for personalized immunotherapy strategies.

The shift toward multi-omic profiling and real-time bioinformatics is crucial for achieving personalized immunotherapy. By characterizing tumors according to their DNA repair status, AR dependency, and immune cell infiltration patterns, clinicians can identify rational combination regimens that optimize efficacy while minimizing unnecessary toxicity. If standardized and validated, liquid biopsies could further refine this process by detecting emergent resistance in near-real time, prompting rapid therapeutic modifications [125]. Figure 4 schematically illustrates how precision medicine approaches using predictive biomarkers and multi-omics analyses can be incorporated into future cancer immunotherapy strategies, suggesting the future direction of personalized treatment.

However, the feasibility of such precision strategies hinges on equitable access to sophisticated testing platforms and the capacity of healthcare systems to absorb the associated financial and logistical demands. Moreover, repeated biopsies or frequent imaging can impose a non-negligible psychological burden on patients. Balancing these challenges with the potential to improve the outcomes remains a key ethical and economic consideration [108].

This figure illustrates a precision medicine approach that integrates existing and emerging predictive biomarkers (e.g., MSI-H/dMMR, CDK12 mutations, TMB, etc.) with multi-omics (genome, transcriptome, proteome, metabolome) and liquid biopsy technology to suggest future directions for cancer immunotherapy. The upper part shows the impact of each biomarker on predicting immunotherapy response, and the lower part presents a flow chart of personalized treatment strategies (e.g., hormone therapy + immunotherapy, radiation + immunotherapy, etc.) based on this molecular information, offering a vision for the future of precision medicine that can be utilized for patient classification and optimal treatment decisions. Created with BioRender.com.

### 6.3. Innovation in Clinical Trial Design

Although conventional randomized controlled trials are indispensable, they may struggle to capture the intricate and evolving nature of immunotherapy. Adaptive designs, where prespecified modifications occur based on interim biomarker data, show promise for more rapid identification of winning strategies and the de-escalation of ineffective ones. Basket trials, already used in other tumor types to evaluate therapies against specific molecular markers, offer an efficient route to test immunotherapies in prostate cancers with features such as dMMR [126].

Collaboration across disciplines is vital; immunologists, geneticists, pathologists, and data scientists must work alongside oncologists to interpret multifaceted trial outcomes. Single-cell RNA sequencing, multiplex immunohistochemistry, and computational pathology can yield transformative insights into treatment responses but require cross-specialty expertise to achieve meaningful clinical applications. Ultimately, these innovative trial frameworks, bolstered by a spirit of collaboration, may help expedite the long-awaited breakthrough in prostate cancer immunotherapy, transcending the current incremental gains and ushering in a new era of personalized and effective treatment options [127].

## 7. Conclusions

Prostate cancer, poised at the intersection of androgen-driven biology and an inherently immunosuppressive TME, has often been labeled an “immune desert”. However, emerging evidence—from the success of sipuleucel-T to the incremental progress of ICIs—demonstrates that immune-based strategies can reshape disease trajectories [128]. In this review, we outlined how checkpoint blockade, ICD inducers, tumor vaccines, CAR T-cell engineering, and various other approaches can potentially convert prostate cancer from a “cold” to a “hot” tumor, thereby enhancing its responsiveness to immunotherapy. By elucidating how antigen presentation, CD8+ T-lymphocyte activation, and proinflammatory cytokine release are interconnected with clinical outcomes, we can gain valuable insights into the design of more potent immunotherapies.

Despite these advances, prostate cancer immunotherapy remains far from achieving routine clinical success. A prominent concern is the incidence of irAEs, which occur frequently and include severe toxicity. When unchecked, the immune activation required for tumor control risks collateral damage to healthy tissues, manifesting as hepatitis, pneumonitis, colitis, and other organ-specific inflammatory conditions. Recognizing the burden of these toxicities is crucial; current guidelines recommend discontinuing ICIs at grade ≥ 2 irAEs and initiating prompt management [120]. Future clinical trials must, therefore, balance efficacy with safety, particularly as combination regimens—hormonal therapy, chemotherapy, radiotherapy, or targeted agents—are introduced to boost immunogenicity but may have adverse effects.

Looking ahead, the field’s trajectory is marked by a collective push toward biomarker-driven individualized care. Multi-omic profiling and real-time liquid biopsies not only illuminate the underlying biology of prostate cancer but also help clinicians identify ideal time points and patient subsets for immunotherapy. Additionally, cutting-edge technologies such as “armored” CAR T cells built to resist immunosuppressive signals, CRISPR-based gene editing for optimizing cellular therapies, and AI systems for predictive modeling each carry the promise of overcoming current resistance mechanisms. If these innovations can be safely and cost-effectively operationalized, prostate cancer may eventually mirror other malignancies that exhibit transformative responses to immunotherapy.

Ultimately, the challenge lies in carefully orchestrating immune activation without compromising patient safety while recognizing the profound heterogeneity within prostate cancer populations. Addressing this balance requires sustained collaborative research integrating molecular oncology, immunology, and clinical trial innovation. By pursuing this multipronged strategy, we may decisively expand the therapeutic window for patients with advanced or metastatic diseases, offering a pathway toward more durable disease control and improved quality of life.

## Figures and Tables

**Table 2:** Summary of major immunotherapy strategy-associated clinical trials in prostate cancer.

Strategy	Selected Clinical Trials	Phase	Population (Patients)	Status/Key Findings
Immune checkpoint blockade				
	CheckMate 650 (NCT02985957)	II	mCRPC	Limited efficacy; subgroup activity in selected biomarkers
	KEYNOTE-199 (NCT02787005)	II	mCRPC	Modest response (~5–10%); better in MSI-H/dMMR patients
	IMbassador250 (NCT03016312)	II	mCRPC (post abiraterone)	No significant OS benefit; better PFS in PD-L1/CD8-high subset
	CONTACT-02 (NCT04446117)	III	mCRPC (after novel hormonal therapy)	Improved PFS; no OS benefit overall; better results in liver/bone metastases
Induction of ICD				
	Docetaxel + vaccine combos (e.g., NCT02649855)	I/II	mHSPC	Mixed immunological outcomes; larger trials needed
TME reversal (“cold-to-hot”)				
	CSF-1R inhibitors (pexidartinib/NCT02472275)	I	Intermediate/high-risk PCa	Improved T-cell infiltration; combination trials ongoing
Tumor vaccines				
	IMPACT (sipuleucel-T/NCT00065442)	III	mCRPC	Improved survival; delayed responses typical
	PROSPECT (PROSTVAC/NCT01322490)	III	mCRPC	No significant survival benefit
	mRNA vaccine (BNT112/NCT04382898)	I/II	mCRPC and high-risk localized	Promising immunogenicity; ongoing
Bispecific T-cell engagers (BiTEs)				
	Xaluritamig (AMG 509/NCT04221542)	I→III	mCRPC (heavily pretreated)	Early promising PSA responses; notable CRS events; phase III planned
Immune adjuvants				
	SD-101 + pembrolizumab + RT + ADT/NCT03007732)	II	Hormone-naïve oligometastatic PCa	Enhanced immune response; moderate efficacy observed
CAR T-cell therapy				
	PSCA-directed CAR T/NCT0387380	I	mCRPC	Early phase; promising but limited responses; ongoing
Interventions targeting stromal barriers				
	COSMIC-021 (cabozantinib + atezolizumab/NCT03170960)	I/II/III	mCRPC	Improved immune infiltration; transient clinical benefits

Abbreviations: ADT, androgen deprivation therapy; BiTE, bispecific T-cell engager; CAR, chimeric antigen receptor; CRS, cytokine release syndrome; CSF-1R, colony-stimulating factor-1 receptor; dMMR, deficient mismatch repair; ICD, immunogenic cell death; mCRPC, metastatic castration-resistant prostate cancer; mHSPC, metastatic hormone-sensitive prostate cancer; MSI-H, microsatellite instability high; OS, overall survival; PCa, prostate cancer; PD-L1, programmed death ligand-1; PFS, progression-free survival; PSCA, prostate stem cell antigen; PSA, prostate-specific antigen; RT, radiation therapy; TME, tumor microenvironment.

**Table 3:** Overview of immunotherapy strategies in prostate cancer.

Approach	Mechanism	Representative Agents or Examples	Key Clinical Findings	Limitations/ Challenges	References
Checkpoint inhibitors	Block inhibitory receptors (PD-1, CTLA-4) on T cells	Nivolumab, ipilimumab	Effective in MSI-H subset	Limited overall response	[38,39]
Induction of ICD	Promote release of DAMPs; enhance antigen presentation and DC activation	Docetaxel, SBRT	May synergize with ICIs	Inconsistent biomarkers	[52,53]
TME reversal	Modify the immunosuppressive milieu; shift M2-like TAMs to an M1 phenotype; reduce Treg and MDSC infiltration	CSF-1R inhibitors	Improved T-cell infiltration	Multiple targets needed	[59,60]
Tumor vaccines	Present tumor-specific antigens (e.g., PSA, PAP) to prime T cells; elicit long-term adaptive immunity	Sipuleucel-T	Improved survival	Antigen escape possible	[65,66,67]
CAR T-cell therapy	Genetically engineer T cells to target prostate-specific antigens (e.g., PSMA, PAP); potential for high specificity	PSMA-targeted CAR	Early responses observed	Toxicity and high cost	[68]
Interventions targeting stromal barriers	Alter tumor stroma/fibrosis to allow for T-cell penetration; normalize vasculature for better immune cell and drug delivery	TGF-β inhibitors	Enhanced immune infiltration	Risk of damaging healthy tissue	[78]

Abbreviations: CAR, chimeric antigen receptor; CSF-1R, colony-stimulating factor-1 receptor; CTLA-4, cytotoxic T-lymphocyte antigen 4; DAMP, damage-associated molecular pattern; DC, dendritic cell; ICD, immunogenic cell death; ICI, immune checkpoint inhibitor; MDSC, myeloid-derived suppressor cell; MSI-H, microsatellite instability high; PAP, prostatic acid phosphatase; PD-1, programmed cell death protein-1; PSA, prostate-specific antigen; PSMA, prostate-specific membrane antigen; SBRT, stereotactic body radiation therapy; TAM, tumor-associated macrophage; TGF-β, transforming growth factor-beta; TME, tumor microenvironment; Treg, regulatory T cell.

**Table 4:** Potential predictive biomarkers for immunotherapy response in prostate cancer.

Biomarker	Detection Method	Clinical Significance	Limitations/Challenges	References
PD-L1 expression	IHC	Partial predictive value	Generally low expression	[90,92]
MSI-H/dMMR	NGS, IHC	Predicts checkpoint inhibitor response	Rare in prostate cancer	[93,95]
CDK12 alterations	NGS panels	Associated with neoantigen load	Requires validation	[91]
TMB	Whole-exome sequencing	Indicates tumor immunogenicity	Lack of standardized cutoffs	[89]
DNA repair mutations	NGS	Facilitates targeted therapy	Variability in response	[104,105]
Liquid biopsy (ctDNA, CTC)	Blood-based analysis	Tracks resistance in real time	Sensitivity/specificity issues	[97,98]

Abbreviations: CDK12, cyclin-dependent kinase 12; ctDNA, circulating tumor DNA; CTC, circulating tumor cell; dMMR, deficient mismatch repair; IHC, immunohistochemistry; MSI-H, microsatellite instability high; NGS, next-generation sequencing; PD-L1, programmed death ligand-1; TMB, tumor mutational burden.

**Table 5:** Combination therapy strategies in prostate cancer immunotherapy.

Combination Approach	Mechanism/Rationale	Representative Trials or Evidence	Key Outcomes/ Observations	Challenges	References
Hormonal + immunotherapy	Transiently enhances T-cell infiltration by modulating AR signaling; may upregulate tumor antigens	ADT + ICIs	Improved T-cell infiltration	Optimal timing unclear	[39,110]
Chemo + immunotherapy	Certain chemotherapeutics (e.g., docetaxel) induce immunogenic cell death; potentially increase antigen release	Docetaxel + ICIs	Enhanced immune responses	Variable ICD induction	[111,112]
Radiotherapy + immunotherapy	Radiation can induce local tumor cell death and promote abscopal effects; releases tumor antigens for DC activation	SBRT + ICIs	Potential abscopal effect	Inconsistent results	[113,114]
PARP inhibitors + immunotherapy	DNA repair inhibition elevates tumor mutational load; may increase neoantigen presentation	Olaparib + ICIs	Efficacy in DNA repair-deficient patients	Limited to certain mutations	[104]
TME-targeting agents + immunotherapy	Disrupt immunosuppressive networks (MDSCs, Tregs, TAMs) to facilitate T-cell infiltration	IDO, TGF-β inhibitors + ICIs	Improved T-cell activity	Complexity of pathways	[60,61,67]
Multi-target regimens	Simultaneously address multiple resistance mechanisms (e.g., PD-1 + CTLA-4 + hormonal + radiotherapy)	PD-1 + CTLA-4 + ADT	Potential increased response	High cumulative toxicity	[67,115]

Abbreviations: ADT, androgen deprivation therapy; AR, androgen receptor; CTLA-4, cytotoxic T-lymphocyte antigen 4; DC, dendritic cell; ICD, immunogenic cell death; ICI, immune checkpoint inhibitor; IDO, indoleamine 2,3-dioxygenase; MDSC, myeloid-derived suppressor cell; PARP, poly (ADP-ribose) polymerase; PD-1, programmed cell death protein-1; SBRT, stereotactic body radiation therapy; TAM, tumor-associated macrophage; TGF-β, transforming growth factor-beta; TME, tumor microenvironment; Treg, regulatory T cell.
